# FANCD2 Binds Human Papillomavirus Genomes and Associates with a Distinct Set of DNA Repair Proteins to Regulate Viral Replication

**DOI:** 10.1128/mBio.02340-16

**Published:** 2017-02-14

**Authors:** Chelsey C. Spriggs, Laimonis A. Laimins

**Affiliations:** Department of Microbiology-Immunology, Northwestern University, Feinberg School of Medicine, Chicago, Illinois, USA; University of Michigan

## Abstract

The life cycle of human papillomavirus (HPV) is dependent on the differentiation state of its host cell. HPV genomes are maintained as low-copy episomes in basal epithelial cells and amplified to thousands of copies per cell in differentiated layers. Replication of high-risk HPVs requires the activation of the ataxia telangiectasia-mutated (ATM) and ATM and Rad3-related (ATR) DNA repair pathways. The Fanconi anemia (FA) pathway is a part of the DNA damage response and mediates cross talk between the ATM and ATR pathways. Our studies show that HPV activates the FA pathway, leading to the accumulation of a key regulatory protein, FANCD2, in large nuclear foci. These HPV-dependent foci colocalize with a distinct population of DNA repair proteins, including ATM components γH2AX and BRCA1, but infrequently with p-SMC1, which is required for viral genome amplification in differentiated cells. Furthermore, FANCD2 is found at viral replication foci, where it is preferentially recruited to viral genomes compared to cellular chromosomes and is required for maintenance of HPV episomes in undifferentiated cells. These findings identify FANCD2 as an important regulator of HPV replication and provide insight into the role of the DNA damage response in the differentiation-dependent life cycle of HPV.

## IMPORTANCE

High-risk human papillomaviruses (HPVs) are the etiological agents of cervical cancer and are linked to the development of many other anogenital and oropharyngeal cancers. Identification of host cellular pathways involved in regulating the viral life cycle may be helpful in identifying treatments for HPV lesions. Mutations in genes of the Fanconi anemia (FA) DNA repair pathway lead to genomic instability in patients and a predisposition to HPV-associated malignancies. Our studies demonstrate that FA pathway component FANCD2 is recruited to HPV DNA, associates with members of the ATM DNA repair pathway, and is essential for the maintenance of viral episomes in basal epithelial cells. Disruption of the FA pathway may result in increased integration events and a higher incidence of HPV-related cancer. Our study identifies new links between HPV and the FA pathway that may help to identify new therapeutic targets for the treatment of existing HPV infections and cancers.

## INTRODUCTION

Human papillomaviruses (HPVs) are the causative agents of cervical cancer along with most anogenital and many oropharyngeal cancers (1, 2). Over 200 types of HPV have been identified, and approximately 10 of these, including types 16, 18, and 31, are referred to as high risk due to their association with the development of cancers (3). HPVs infect the basal layer of stratified epithelia and establish their double-stranded DNA genomes as nuclear episomes at approximately 100 copies per cell. Upon epithelial differentiation, HPV-infected cells override cell cycle checkpoint controls to reenter S/G_2_ phase and amplify their genomes to thousands of copies per cell (4, 5). HPV genomes are approximately 8 kb in size and encode eight open reading frames. In infected basal cells, early gene expression is controlled by the p97 promoter, which is regulated by viral and cellular factors through binding at sequences in the viral upstream regulatory region (URR) (6). The early promoter directs transcription of polycistronic messages that encode proteins that contribute to the stable maintenance of HPV genomes, including the E1 and E2 replication proteins and the E6 and E7 viral oncoproteins (7, 8). The late promoter, p742, is activated upon differentiation and controls expression of the L1 and L2 capsid proteins along with E1, E1^E4, E2, and E5, which are involved in regulating genome amplification and late gene expression (9–12).

The productive life cycle of HPV is dependent upon activation of both the ataxia-telangiectasia mutated (ATM) and the ATM and Rad3-related (ATR) DNA repair pathways (13–16). The ATM pathway is activated in response to DNA double-stranded breaks, while ATR responds to replication stress and the presence of single-stranded DNA at stalled replication forks (17, 18). High-risk HPVs have been shown to selectively activate and repress components of these signaling pathways to promote viral replication (19); however, which members of these pathways are involved in regulating episomal maintenance as well as differentiation-dependent genome amplification is still not fully understood.

The Fanconi anemia (FA) pathway cross talks with the ATM and ATR pathways in cell cycle control and the repair of DNA interstrand cross-links (20). Interstrand cross-links are covalent linkages between opposite strands of DNA that are generated by mistakes in replication or the action of DNA-alkylating agents. These toxic lesions block both replication and transcription, making their resolution essential for cell survival (21). The FA pathway is composed of 20 complementation groups, including FANCA, -B, -C, -E, -F, -G, -L, and -M, which together form the FA core complex. Replication stress activates the FA core, leading to monoubiquitination of the FANCD2/FANCI heterodimer through the E3 ubiquitin ligase activity of the FANCL subunit. Monoubiquitinated FANCD2 (FANCD2-Ub) colocalizes with other repair factors, including γH2AX and BRCA1, to facilitate the recruitment of downstream effector proteins for DNA repair (22). ATR directly phosphorylates several proteins in the FA pathway, including FANCD2, which is required for optimal FANCD2 monoubiquitination and the formation of nuclear repair foci (23–25). FANCD2 is also phosphorylated by ATM, but this leads to an S-phase arrest and is not involved in FA pathway-mediated repair (26). 

FA is a rare genetic disorder caused by mutations in one or more genes of the FA pathway. It is characterized by genomic instability, bone marrow failure, and congenital defects (27). Moreover, FA patients have an increased susceptibility to squamous cell carcinomas (SCCs), particularly those of the oral cavity and anogenital regions (28), which are preferred sites of HPV infection. HPV DNA has been detected in over 80% of SCCs from FA patients compared to 36% from control subjects, suggesting that the loss of FA pathway activity promotes viral transformation (29). Additional studies have reported that expression of high-risk E7 is sufficient for FA pathway activation leading to accelerated chromosomal instability in FA cells (30). Furthermore, the loss of FANCA or FANCD2 leads to a posttranscriptional accumulation of E7 and stimulates hyperplastic growth in HPV cells (31, 32).

In this study, we investigated the role of the FA pathway in regulating the HPV viral life cycle and its interactions with members of the ATM/ATR pathway. Our studies show that HPV activates FANCD2 and increases its levels. This results in the accumulation of FANCD2 in distinct nuclear foci, where it colocalizes with other DNA repair factors as well as HPV DNA. We further demonstrate that FANCD2 is preferentially recruited to viral genomes, compared to chromosomal DNA, and plays a critical role in maintaining viral episomes in undifferentiated cells. Our study demonstrates that the FA pathway is an essential regulator of viral replication and is associated with specific populations of DNA repair factors during the differentiation-dependent viral life cycle.

## RESULTS

### FANCD2 protein levels are increased in HPV-positive cells.

**FIG 1:**
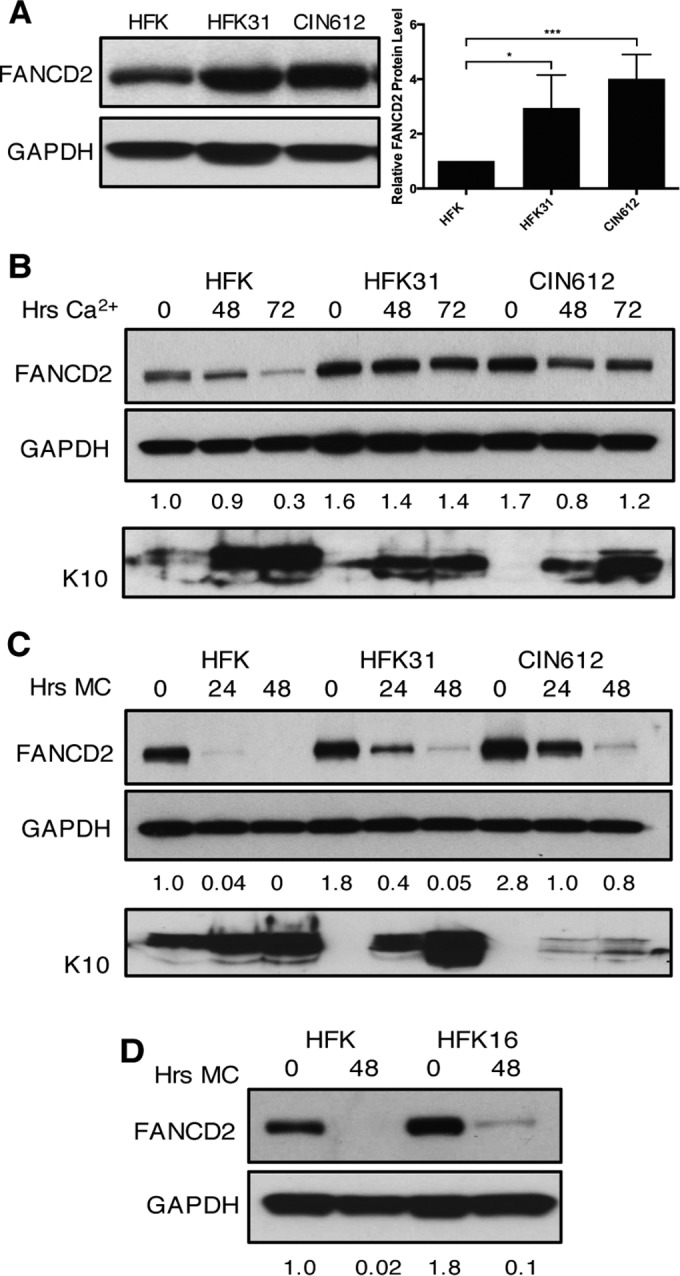
Levels of FANCD2 are increased in HPV-positive cells and remain elevated through differentiation. (A) Western blot analysis of FANCD2 levels in normal human foreskin keratinocytes (HFKs), HFKs stably transfected with HPV31 whole genomic DNA (HFK31), and CIN612 cells. The graph demonstrates FANCD2 protein levels relative to GAPDH and normalized to FANCD2 levels in HFKs across three independent experiments. Error bars represent standard deviations between experiments. A standard Student’s *t* test was used to determine statistical significance. *, *P* ≤ 0.05; ***, *P* ≤ 0.001. (B) Western blot analysis of FANCD2 levels in HFK, HFK31, and CIN612 cells that were differentiated in 1.5 mM calcium medium for 48 or 72 h. Epithelial differentiation was confirmed by levels of cytokeratin 10. (C) Western blot analysis of FANCD2 levels in HFK, HFK31, and CIN612 cells that were differentiated in 1.5% methylcellulose for 24 or 48 h. Epithelial differentiation was confirmed by levels of cytokeratin 10. (D) Western blot analysis of FANCD2 levels in HFK and HFKs stably transfected with HPV16 DNA that were differentiated for 48 h in 1.5% methylcellulose. Quantification of FANCD2 band intensity was determined by densitometry using Image Lab software relative to GAPDH and normalized to HFKs.

To investigate the role of the FA pathway in the HPV life cycle, we first examined the levels of FANCD2 in normal and HPV31-positive cells. Western blot analysis was performed on cell extracts isolated from primary human foreskin keratinocytes (HFKs) along with extracts from HFKs that had been stably transfected with whole genomic HPV31 DNA (HFK31) or CIN612 cells, an immortalized, patient-derived cell line that stably maintains HPV31 episomes. This analysis revealed a statistically significant increase in the levels of FANCD2 in HPV-positive cells compared to control HFKs (Fig. 1A). As the life cycle of HPV is closely linked to the differentiation state of its host cell, we next investigated if FANCD2 levels are altered in HPV-positive cells upon epithelial differentiation induced by the addition of medium containing 1.5 mM calcium. Upon differentiation, the levels of FANCD2 declined rapidly in uninfected control cells, while in HPV-positive cells, FANCD2 was maintained at high levels through differentiation (Fig. 1B). Cellular differentiation was confirmed using cytokeratin 10, an intermediate filament protein found in suprabasal epithelial cell layers. We also examined cellular differentiation by suspension in 1.5% methylcellulose and saw retention of FANCD2 levels in HPV31-positive cells compared to HFK control cells, although levels decreased moderately (Fig. 1C), as well as in HFKs that stably maintained episomal copies of transfected high-risk HPV16 DNA (Fig. 1D).

### Large FANCD2 foci form in HPV-positive cells that become more numerous upon differentiation.

**FIG 2:**
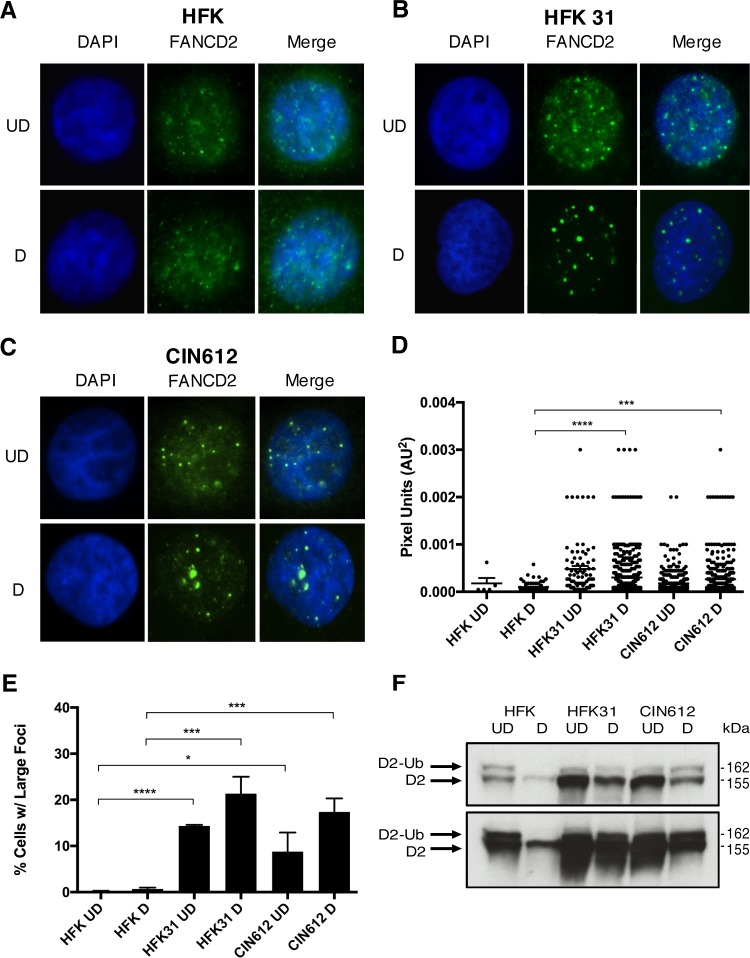
HPV infection leads to FA pathway activation. (A to C) Immunofluorescence analysis of FANCD2 localization in HFK, HFK31, and CIN612 cells that were differentiated for 72 h in 1.5 mM calcium medium. Cells were stained with anti-FANCD2 (green) and counterstained with DAPI (blue). UD, undifferentiated; D, differentiated. (D) ImageJ software was used to quantitate focus size by automated particle analysis. The graph represents the size of individual foci represented in pixel units (AU^2^). Error bars represent the standard errors of the means within the sample. A standard Student’s *t* test was used to determine statistical significance. ***, *P* ≤ 0.0005; ****, *P* ≤ 0.0001. Differences in focus size between undifferentiated cell populations were not statistically significant. (E) The graph demonstrates the percentage of cells with large, nuclear FANCD2 foci. Error bars represent the standard deviations between experiments. A standard Student’s *t* test was used to determine statistical significance. *, *P* ≤ 0.05; ***, *P* ≤ 0.0005; ****, *P* ≤ 0.0001. (F) Western blot analysis of FANCD2-Ub (D2-Ub) in HFK, HFK31, and CIN612 cells that were differentiated for 72 h in 1.5 mM calcium medium (top). A longer exposure shows that FANCD2-Ub is undetectable in differentiated HFK samples (bottom).

Activation of the FA pathway induces monoubiquitination of FANCD2, which results in the formation of distinct nuclear repair foci (33). As the formation of nuclear foci is often used as an indicator of FANCD2 activation, we differentiated cells for 72 h in medium containing 1.5 mM calcium and performed immunofluorescence on HPV-positive and -negative cells to assess FANCD2 localization. FANCD2 foci were observed in both control (Fig. 2A) and HPV-positive cells (Fig. 2B and C); however, larger and more discrete foci were detected in HPV-positive cells that were not visible in HFK control cells (Fig. 2D). The sizes of foci were measured using automated particle analysis in ImageJ. While these large foci were often observed in both undifferentiated and differentiated cells, the percentage of cells containing these larger foci and the number of foci per cell increased with differentiation of HPV-positive cells (Fig. 2E). To further confirm FA pathway activation, we performed Western blot analysis to screen for the presence of monoubiquitinated FANCD2 (FANCD2-Ub) in these cells. FANCD2-Ub can be distinguished from the unmodified protein through a slight upward shift in mobility (33). Similar to our immunofluorescence results, FANCD2-Ub was observed in undifferentiated HFKs as well as HPV-positive cells at similar levels. Upon differentiation, HPV-positive cells maintained high levels of the active, monoubiquitinated form of FANCD2, while the levels of FANCD2-Ub in HFKs decreased and were below the level of detection (Fig. 2F).

**FIG 3:**
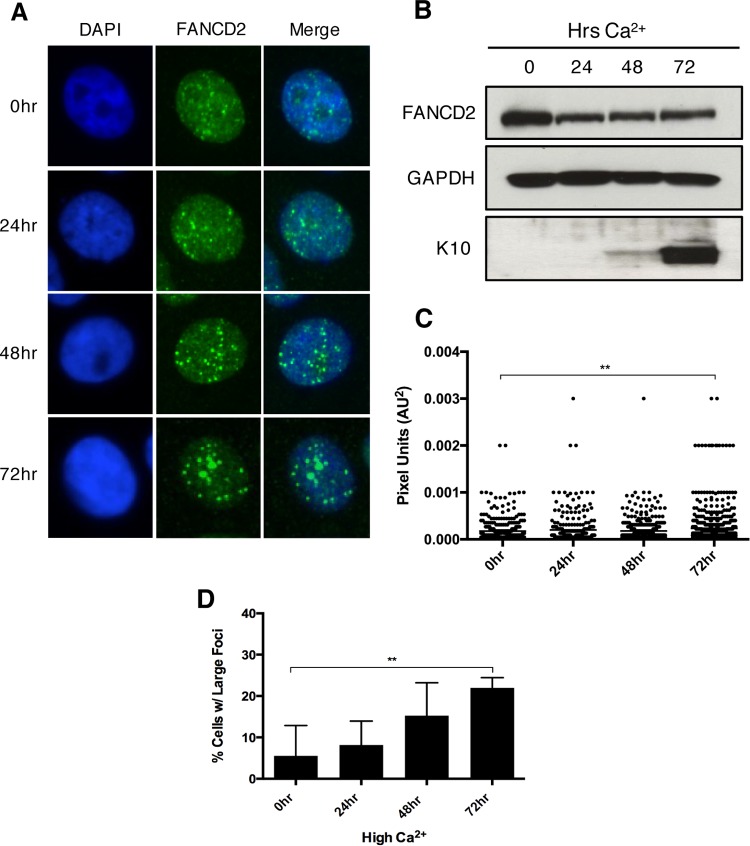
FA pathway activation further increases as differentiation progresses in HPV-positive cells. (A) Immunofluorescence analysis of FANCD2 localization in CIN612 cells that were differentiated in 1.5 mM calcium for 24, 48, or 72 h. Cells were stained with anti-FANCD2 (green) and counterstained with DAPI (blue). (B) Western blot analysis of FANCD2 levels in CIN612 cells that were differentiated in high-calcium medium for 24, 48, or 72 h. GAPDH was used as a loading control. Epithelial differentiation was confirmed by levels of cytokeratin 10. (C) ImageJ software was used to quantitate focus size by an automated particle analysis program. The graph represents individual focus size represented in pixel units (AU^2^). Error bars represent the standard error mean within the sample. A standard Student’s *t* test was used to determine statistical significance. **, *P* ≤ 0.005. (D) The graph demonstrates the percentage of cells with large nuclear FANCD2 foci. Error bars represent the standard deviations between experiments. A standard Student’s *t* test was used to determine statistical significance. **, *P* ≤ 0.005.

To establish when during cellular differentiation these large FANCD2 foci begin to form, immunofluorescence was used to visualize FANCD2 localization in CIN612 cells that were induced to differentiate in high-calcium medium for 24, 48, or 72 h (Fig. 3A). While large FANCD2 foci were observed in undifferentiated cells (Fig. 3C), the percentage of cells with large foci increased after 48 h and continued until 72 h (Fig. 3D). This coincides with induction of cytokeratin 10 (Fig. 3B) and correlates with the initiation of HPV amplification in cells, which begins 48 h after a calcium switch and increases through 72 h (13). Together, these results indicate that HPV activates the FA pathway and induces a redistribution of FANCD2 into large nuclear foci. Interestingly, these foci form more frequently upon differentiation, compared to undifferentiated cells, despite lower levels of total FANCD2 in these cells.

### FANCD2 colocalizes with a distinct population of DNA repair proteins in nuclear foci.

**FIG 4:**
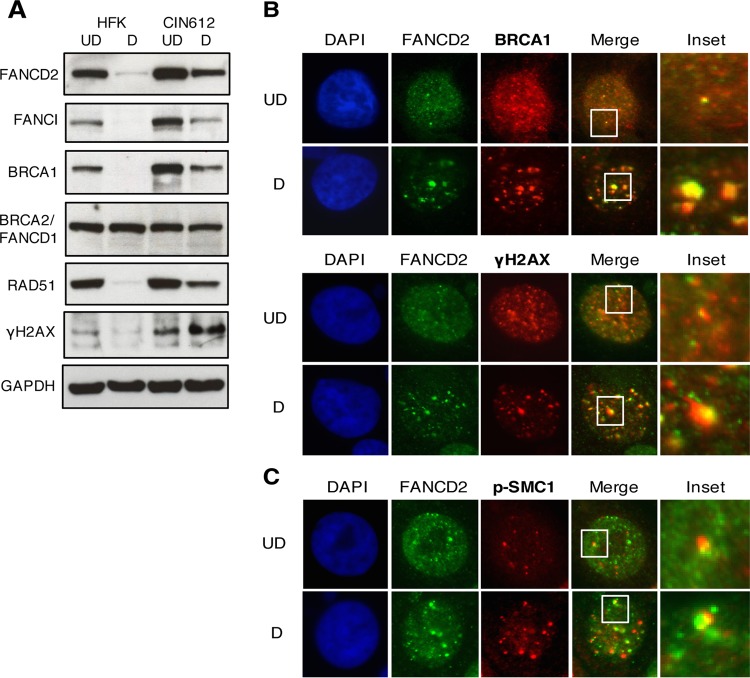
FANCD2 colocalizes with components of the ATM pathway in discrete nuclear foci. (A) HFKs and CIN612 cells were differentiated for 72 h in 1.5 mM calcium medium. Western blot analysis was performed using antibodies to FANCD2, FANCI, BRCA1, BRCA2, RAD51, and γH2AX. GAPDH was used as a loading control. (B and C) CIN612 cells were differentiated for 72 h in 1.5 mM calcium medium and stained with anti-FANCD2 (green) and either anti-BRCA1, anti-γH2AX, or anti-p-SMC1 (red). Cells were counterstained with DAPI (blue). UD, undifferentiated; D, differentiated.

To investigate whether other proteins involved in DNA repair are localized to FANCD2 nuclear foci in HPV-positive cells, we examined the levels of several FA-associated proteins by Western blot analysis (Fig. 4A). The levels of FANCI, BRCA1, and γH2AX were increased in HPV-positive cells, and these proteins were retained at high levels during differentiation compared to control HFKs. A slight increase of approximately 40% was seen in RAD51 levels in undifferentiated HPV-positive cells, and RAD51 was retained at elevated levels during differentiation. The levels of BRCA2/FANCD1 were similar between cell types. These results are consistent with previous findings that HPV induces a DNA damage response that is maintained throughout the differentiation-dependent viral life cycle (13). BRCA1 and γH2AX are intricately involved in FA pathway repair as BRCA1 colocalizes with FANCD2 at sites of damage and γH2AX is required for recruiting FANCD2 to chromatin at stalled replication forks (33, 34). To determine whether FANCD2 colocalizes with these factors in HPV-positive cells, we performed coimmunofluorescence for FANCD2 with BRCA1 or γH2AX. BRCA1 and γH2AX were found to colocalize with FANCD2 in large as well as small foci, in both undifferentiated and differentiated cells (Fig. 4B). While we did not perform confocal microscopy to deconvolute these images, we believe that the overlap we observe is likely indicative of colocalization. Overall, our studies suggest that FANCD2 localizes to nuclear foci in HPV-positive cells that may be sites of DNA repair.

**FIG 5:**
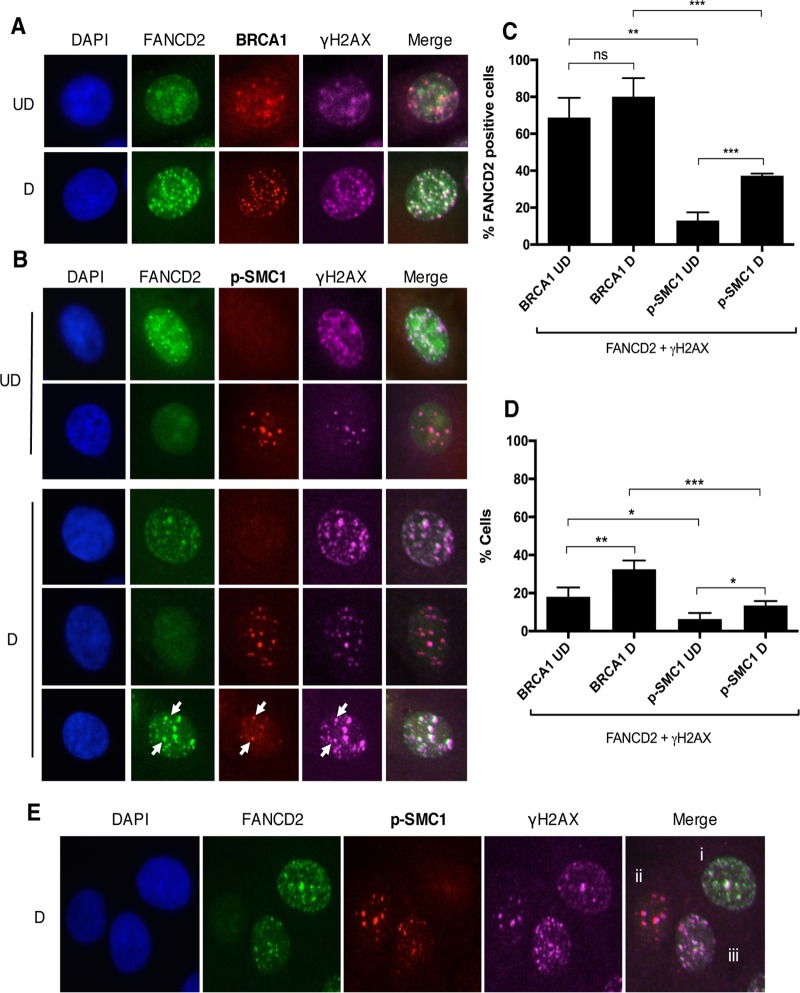
Distinct populations of foci exist during HPV infection. (A and B) CIN612 cells were differentiated for 72 h in 1.5 mM calcium medium. Immunofluorescence analysis was performed on cells stained with anti-FANCD2 (green) and either anti-BRCA1 or anti-p-SMC1 (red). Cells were then counterstained with anti-γH2AX (pink) and DAPI (blue). Arrows indicate foci where FANCD2, γH2AX, and p-SMC1 are found together. UD, undifferentiated; D, differentiated. (C) The graph demonstrates the percentage of cells with FANCD2 foci where at least one focus colocalizes with γH2AX and either BRCA1 or p-SMC1. (D) The graph represents the percentage of all HPV-positive cells where at least one FANCD2 focus colocalizes with γH2AX and either BRCA1 or p-SMC1. Error bars represent the standard deviations between experiments. A standard Student’s *t* test was used to determine statistical significance. *, *P* ≤ 0.05; **, *P* ≤ 0.01; ***, *P* ≤ 0.001. ns, not significant. (E) Representative image of three distinct populations in differentiated CIN612 cells stained with anti-FANCD2 (green), anti-p-SMC1 (red), anti-γH2AX (pink), and DAPI (blue). Populations are identified as having FANCD2 foci with no p-SMC1 foci (i), having p-SMC1 foci with no FANCD2 foci (ii), and having both FANCD2 and p-SMC1 foci (iii).

BRCA1 and γH2AX also form complexes with p-SMC1, a cohesin protein that plays a role in G_2_/M cell cycle arrest as well as DNA homologous recombination repair (35, 36). Previously, p-SMC1 was identified as an important regulator of the HPV life cycle and essential for differentiation-dependent genome amplification (37). Using immunofluorescence, we investigated if FANCD2 also colocalizes with p-SMC1 in HPV-positive cells. Interestingly, while FANCD2 and p-SMC1 were occasionally found in the same nucleus, they were rarely colocalized into the same foci (Fig. 4C). Since FANCD2 is found to colocalize with BRCA1 and γH2AX, but not with p-SMC1, we investigated whether different populations of repair foci exist in HPV-positive cells. For this analysis, 4-color immunofluorescence was used to determine if FANCD2 colocalizes with the same population of γH2AX as BRCA1 and p-SMC1. In the majority of cells with FANCD2-positive nuclear foci, FANCD2 colocalized with BRCA1 and γH2AX (68.8% ± 6.145%), and this population increased modestly (80.09% ± 5.028%), but not significantly, with cellular differentiation (Fig. 5A and C). In contrast, FANCD2 was infrequently found to colocalize with p-SMC1 (13.08% ± 2.551%) (Fig. 5B and C). Cells with FANCD2 nuclear foci were found to have low p-SMC1 signals, and cells containing p-SMC1 foci exhibited low levels of FANCD2. Interestingly, both FANCD2 and p-SMC1 foci also contained γH2AX, but in different populations of cells. A small subset of cells was identified in which FANCD2 and p-SMC1 were present in the same foci, but this group represented less than 14% of the total cell population and usually had only one or two positive foci (Fig. 5B and D). These findings indicate that there are at least three distinct populations of HPV-positive cells, which can be characterized by the DNA repair proteins localized within them: (i) those that are FANCD2 positive and p-SMC1 negative, (ii) those that are p-SMC1 positive and FANCD2 negative, and (iii) a smaller subset in which FANCD2 and p-SMC1 foci are found together (Fig. 5E).

### FANCD2 preferentially binds HPV DNA compared to cellular DNA.

**FIG 6:**
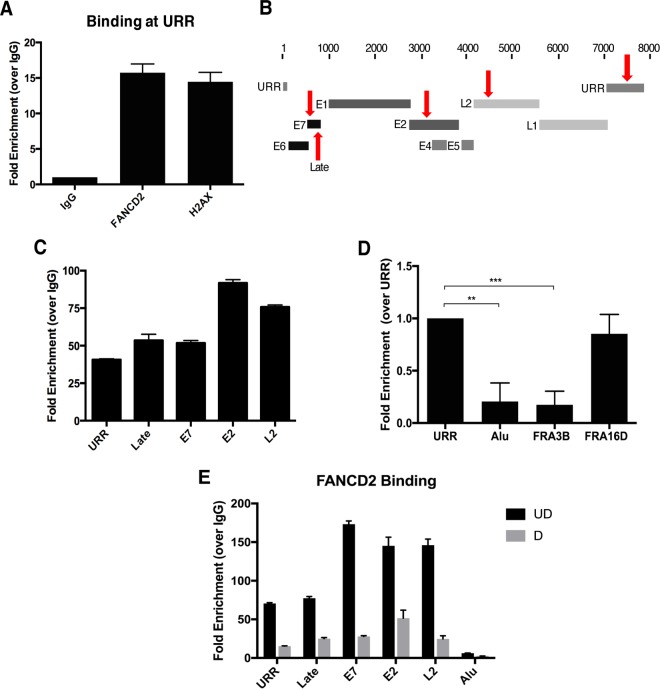
FANCD2 is preferentially recruited to HPV DNA. (A) Chromatin immunoprecipitation (ChIP) analysis of FANCD2 and γH2AX binding to the URR in CIN612 cells. Quantitative real-time PCR (qRT-PCR) was performed using a LightCycler 480 (Roche), and fold enrichment was quantitated relative to an IgG control. Similar results were seen in three independent experiments. Error bars represent the standard deviations between experiments. (B) Schematic of the HPV31 linearized genome, with primer regions indicated with arrows. (C) ChIP analysis for FANCD2 binding at indicated sites in the viral genome. Fold enrichment was normalized to an IgG control. Similar results were seen in three independent experiments. Error bars represent the standard deviations between experiments. (D) ChIP analysis of FANCD2 binding at the URR compared to Alu repeat and fragile site regions (FRA3B and FRA16D) in the host genome. Enrichment was normalized to an IgG control and is represented as fold change over URR across three independent experiments. The graph represented as percentage of input shows a similar trend (Fig. S1). Error bars represent the standard deviations between experiments. A standard Student’s *t* test was used to determine statistical significance. **, *P* < 0.005; ***, *P* < 0.0005. (E) CIN612 cells were differentiated for 72 h in 1.5 mM calcium medium, and ChIP analysis was performed for binding across the HPV genome. Fold enrichment was normalized to an IgG control. Similar results were seen in three independent experiments. Error bars represent the standard deviations between experiments. UD, undifferentiated; D, differentiated.

DNA damage factors, including γH2AX and p-SMC1, have been shown to bind to HPV genomes (37, 38). As FANCD2 is associated with γH2AX in HPV-positive cells, we used chromatin immunoprecipitation (ChIP) to determine whether FANCD2 also binds viral genomes. We first assessed FANCD2 binding at the URR and found that, like γH2AX, FANCD2 bound to this region (Fig. 6A). To determine whether FANCD2 binding was specific to the URR, binding was also assessed at other regions along the viral genome (Fig. 6B). In addition to the URR, FANCD2 also was found to bind regions in the late promoter, E7, E2, and L2, suggesting that FANCD2 binds uniformly along the HPV genome (Fig. 6C). To determine if there is a differential recruitment of FANCD2 to viral or cellular genomes, FANCD2 binding to the URR was compared to binding at cellular DNA using the multicopy Alu repeat sequence as a representative cellular locus. FANCD2 binding also was compared to two previously identified fragile sites in the human genome that are often associated with FANCD2—FRA3B and FRA16D (39, 40). Fragile sites are chromosomal regions that are prone to genomic instability during replication stress and are often enriched for DNA repair factors, as they are susceptible to spontaneous breakage (41, 42). We found that FANCD2 bound to HPV DNA to a similar degree to fragile site FRA16D and nearly 10-fold higher than to control Alu repeat regions or to FRA3B (Fig. 6D). A similar trend was observed when represented as a percentage of input (see Fig. S1 in the supplemental material). These results suggest that FANCD2 is preferentially recruited to HPV genomes compared to host cellular DNA. To determine how cellular differentiation affects FANCD2 recruitment to the HPV genome, HPV-positive cells were differentiated for 72 h in high-calcium medium, and FANCD2 binding at viral DNA was examined by ChIP. Interestingly, upon differentiation, binding to viral sequences was reduced by 3- to 6-fold compared to that in undifferentiated cells (Fig. 6E). These results indicate that preferential binding of FANCD2 to viral genomes occurs in undifferentiated cells, and this decreases upon differentiation.

10.1128/mBio.02340-16.1 FIG S1  FANCD2 is preferentially recruited to HPV DNA. ChIP analysis of FANCD2 binding at the URR compared to Alu repeat and fragile site regions in the host genome. The graph represents the fold change over the URR across three independent experiments. All percentage of input data are enriched at least 2-fold over an IgG control. Error bars represent the standard deviations between experiments. A standard Student’s *t* test was used to determine statistical significance. *, *P* < 0.05; ****, *P* < 0.0001. Download FIG S1, TIF file, 0.2 MB. Copyright © 2017 Spriggs and Laimins. 2017 Spriggs and Laimins This content is distributed under the terms of the Creative Commons Attribution 4.0 International license.

### FANCD2 is localized to viral replication centers.

**FIG 7:**
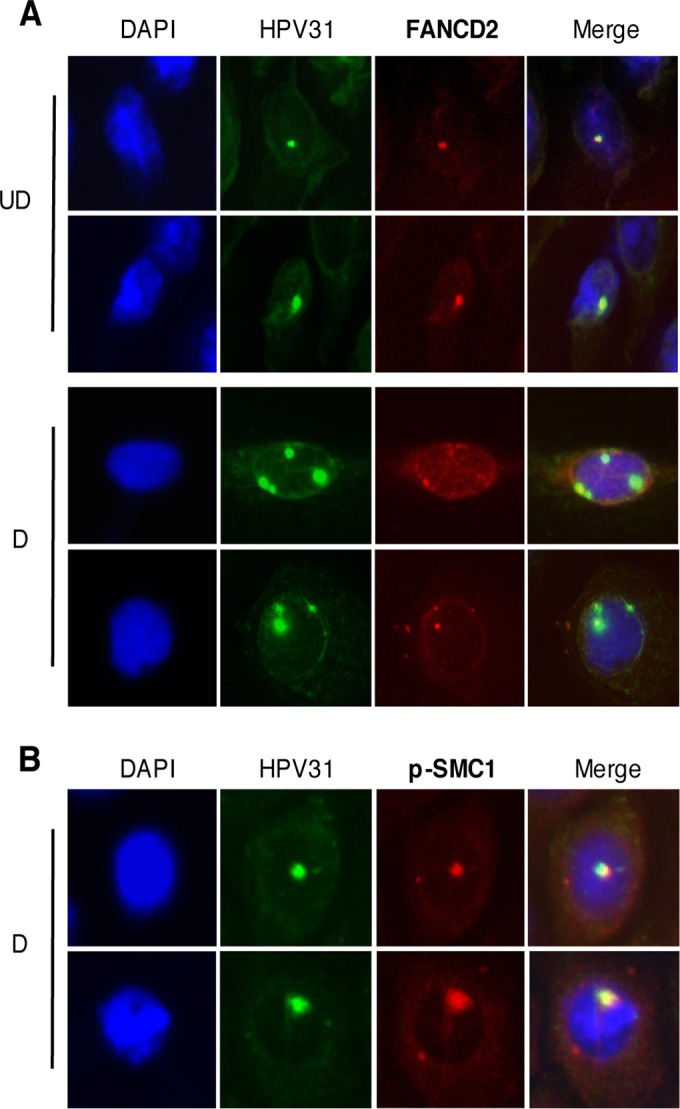
FANCD2 localizes to HPV replication centers. (A) CIN612 cells were differentiated for 72 h in 1.5 mM calcium medium. Immunofluorescence analysis for FANCD2 (red) was performed followed by fluorescent *in situ* hybridization (I-FISH) for HPV31 DNA (green). Cells were counterstained with DAPI (blue). In undifferentiated cells, the FANCD2 signal overlapped with 42.47% ± 12.17% of the HPV31 DNA signal and 11.55%± 1.479% in differentiated cells. UD, undifferentiated; D, differentiated. (B) CIN612 cells were differentiated in 1.5 mM calcium medium, and immunofluorescence analysis for p-SMC1 (red) was performed followed by fluorescent *in situ* hybridization for HPV31 DNA (green). Cells were counterstained with DAPI (blue). In differentiated cells, the p-SMC1 overlapped with 31.85% ± 8.54% of the HPV31 DNA signal. The percentage of overlap between the HPV31 DNA signal and either FANCD2 or p-SMC1 was measured using ImageJ area analysis and found to be statistically significant where *P* is <0.05.

HPV genomes localize at distinct nuclear foci, referred to as viral replication centers, which also contain cellular repair and homologous recombination factors (38). As FANCD2 is preferentially recruited to HPV DNA, we next wanted to determine whether it is associated with viral genomes in these foci. For this analysis, immunofluorescence for FANCD2 was first performed, followed by fluorescent *in situ* hybridization (I-FISH) for HPV31 DNA. Previous studies used FISH to show that HPV genomes localize to one or two foci in undifferentiated cells and that the number and size of these foci increase upon differentiation (13). Our studies show that FANCD2 localizes to HPV replication foci in undifferentiated cells. Upon differentiation, FANCD2 colocalizes to a smaller proportion of the total HPV DNA signal than in undifferentiated cells (Fig. 7A). The percentage of overlap between FANCD2 and the total HPV DNA signal was measured using ImageJ area analysis. In undifferentiated cells, the FANCD2 image overlapped with approximately 42% of the HPV DNA signal, but less than 12% in differentiated cells. These results are in agreement with our ChIP data, which show decreased binding of FANCD2 at viral genomes in differentiated cells. In contrast, when I-FISH was performed for p-SMC1 at HPV DNA, p-SMC1 colocalized with viral DNA in differentiated cells (Fig. 7B). This suggests that the presence of FANCD2 or p-SMC1 nuclear foci in HPV-positive cells may indicate whether the cell is amplifying viral genomes or not.

### FANCD2 loss leads to reduced episomal maintenance in undifferentiated cells.

**FIG 8:**
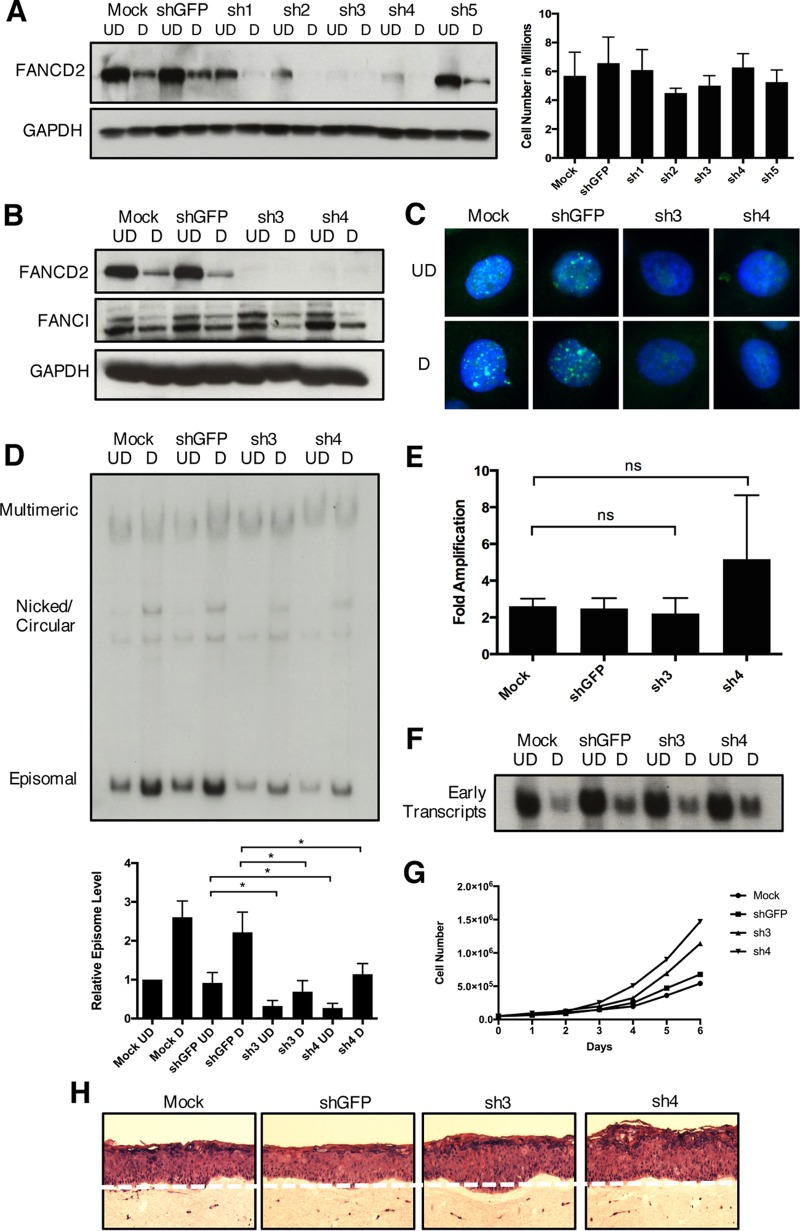
Knockdown of FANCD2 limits HPV31 replication. (A) CIN612 cells were transiently transduced with lentiviral vectors encoding five individual shRNAs against FANCD2 or GFP as a control. After 48 h, cells were differentiated in 1.5% methylcellulose for an additional 48 h. FANCD2 knockdown was assessed by Western blot analysis using GAPDH as a loading control. At 96 h posttransduction, cells were harvested and stained with trypan blue (Bio-Rad) to assess cell viability. The graph shows the total number of live cells in monolayer culture at the time of collection. Error bars represent the standard deviations between measurements. UD, undifferentiated; D, differentiated. (B) CIN612 cells transduced with shGFP or shFANCD2 were differentiated for 48 h in 1.5% methylcellulose, and Western blot analysis was used to assess FANCD2 and FANCI protein levels. GAPDH was used as a loading control. (C) Immunofluorescence analysis of control and FANCD2 knockdown cells that were differentiated for 72 h in 1.5 mM calcium medium. Cells were stained with anti-FANCD2 (green) and counterstained with DAPI (blue). (D) CIN612 cells were differentiated for 48 h in 1.5% methylcellulose, and total DNA was isolated from control and shFANCD2 cells. Viral replication was assessed by Southern blot analysis. Similar results were seen using high calcium concentrations to induce differentiation (Fig. S2). Quantification of episomal band intensity was determined by densitometry using Image Lab software and normalized to the undifferentiated shGFP-infected sample across three independent experiments. Differences in episomal levels between mock- and shGFP-infected cells were not statistically significant. Error bars represent the standard deviations between experiments. A standard Student’s *t* test was used to determine statistical significance. *, *P* ≤ 0.05. (E) The ratio of episomal DNA in undifferentiated and differentiated samples was calculated to determine fold amplification in knockdown and control cells. Error bars represent the standard deviations between experiments. ns, not significant. (F) Control and shFANCD2 cells were differentiated for 48 h in 1.5% methylcellulose. Total RNA was isolated, and early transcript expression was determined by Northern blot analysis. The Northern blot shows expression of the primary early transcript E6*E7 E1^E4 E5. (G) CIN612 cells that stably express either control or shFANCD2 were seeded at 5 × 10^4^ into each well of a 6-well cell culture dish. Cells were harvested and counted each day for 6 days or until reaching confluence. (H) H&E stain of control of shFANCD2-expressing HFK31 cells that were differentiated for 14 days in organotypic raft culture. Similar results were seen in CIN612 cells grown in raft culture.

Our data indicate that FANCD2 is localized to viral replication centers as well as to HPV DNA in undifferentiated cells and colocalizes with proteins essential for HPV replication. To directly examine if FANCD2 has a role in viral replication, CIN612 cells were infected with lentiviral vectors expressing short hairpin RNAs (shRNAs) against either FANCD2 or green fluorescent protein (GFP) as a control. Western blot analysis confirmed that four of five individual shRNAs reduced FANCD2 expression while showing no effect on cell viability (Fig. 8A). We identified two shRNAs, sh3 and sh4, as most effective in reducing protein levels as well as FANCD2 nuclear focus formation, and these were used in subsequent assays (Fig. 8B and C). To determine the effect of FANCD2 knockdown on HPV replication, we first transiently infected CIN612 cells with lentiviruses expressing shRNAs against FANCD2 and screened for its effects on stable maintenance of HPV episomes as well as following differentiation in high-calcium medium by Southern blot analysis. Knockdown of FANCD2 resulted in reduced episomal maintenance in monolayer cells as well as reduced genome amplification upon cellular differentiation (Fig. 8D). Similar results were observed in CIN612 cells stably expressing sh3 and sh4 (see Fig. S2 in the supplemental material). To determine if the loss of FANCD2 directly affects genome amplification, the fold amplification in differentiated cells relative to episomal copy number in monolayer cells was calculated. A similar level of amplification was observed in knockdown cells to that found in control cells (Fig. 8E). This indicates that the reduced levels of amplification in differentiated knockdown cells were primarily the result of impaired episomal maintenance in monolayer cells. From this, we conclude that FANCD2 contributes to episomal maintenance in undifferentiated cells, but minimally to genome amplification in differentiated cells. To determine if FANCD2 regulates episomal maintenance through an effect on early gene expression, total RNA was isolated from control and FANCD2 knockdown cells, and early transcript expression was evaluated by Northern blotting. Interestingly, our data show that the loss of FANCD2 has no effect on HPV early transcript expression (Fig. 8F). This indicates that the FA pathway does not control HPV episomal maintenance indirectly by regulation of viral gene expression. Finally, we investigated if FANCD2 affected the growth and stratification of HPV-positive cells. Compared to control HPV-positive cells, FANCD2 knockdown cells displayed a hyperplastic phenotype when grown in organotypic rafts, as well as a slight growth advantage over time in stable FANCD2 knockdown cells grown in culture (Fig. 8F and G). Taken together, our findings indicate that HPV activates the FA pathway, in part to recruit FANCD2 to viral DNA, where it colocalizes with other DNA repair factors for HPV replication.

10.1128/mBio.02340-16.2 FIG S2  Knockdown of FANCD2 limits episomal maintenance. (A) CIN612 cells were transduced with lentiviral vectors encoding shRNAs against FANCD2 or GFP as a control. After 48 h, cells were treated with 1 μg/ml puromycin until the mock-infected cells died. Cells were then maintained in E-medium with EGF containing 0.5 μg/ml puromycin. Cells were differentiated for 48 h in 1.5% methylcellulose, and FANCD2 levels were assessed by Western blot analysis. GAPDH was used as a loading control. Total DNA was isolated, and viral replication was assessed by Southern blot analysis. (B) Stable knockdown cells were differentiated for 72 h in 1.5 mM calcium medium, and FANCD2 levels were assessed by Western blot analysis. GAPDH was used as a loading control. Total DNA was isolated, and viral replication was assessed by Southern blot analysis. Download FIG S2, TIF file, 4.8 MB. Copyright © 2017 Spriggs and Laimins. 2017 Spriggs and Laimins This content is distributed under the terms of the Creative Commons Attribution 4.0 International license.

## DISCUSSION

The Fanconi anemia pathway is a critical component of the DNA damage response, as it regulates the repair of interstrand cross-links (43). Our studies demonstrate that the levels of FA proteins, including FANCD2, are increased in HPV-positive cells compared to normal keratinocytes. Upon differentiation, the levels of FANCD2 decline rapidly in normal cells, while in HPV-positive cells, higher levels are retained throughout differentiation. Similar increases in FANCD2 levels have been reported in cells expressing either E7 or E6/E7, suggesting that E7 is responsible for these increases (30). Detection of DNA interstrand cross-links induces the monoubiquitination of FANCD2 and the formation of distinct nuclear foci, which are used as a marker for FA pathway activation (33). In our studies, a low level of FANCD2 foci was observed in normal cells; however, significantly higher levels were detected in HPV-positive cells. Interestingly, a population of larger FANCD2 foci was detected in HPV cells, which was not observed in control HFKs. These large foci increase in number upon differentiation and are found in approximately 25% of cells, despite decreases in total levels of FANCD2 protein. These observations demonstrate that the FA pathway is activated in HPV-positive cells, leading to the recruitment of FANCD2 to large nuclear foci. The function of these larger foci in the HPV life cycle is unclear, although we believe it is virus specific, as similar structures have not been reported in studies examining non-virus-induced FA pathway activation. Interestingly, similar activation of the FA pathway and FANCD2 accumulation has been observed in other DNA viruses, including adenovirus, herpes simplex virus 1, and simian virus 40, where it was shown to be important for productive viral replication and growth (44–46).

**FIG 9:**
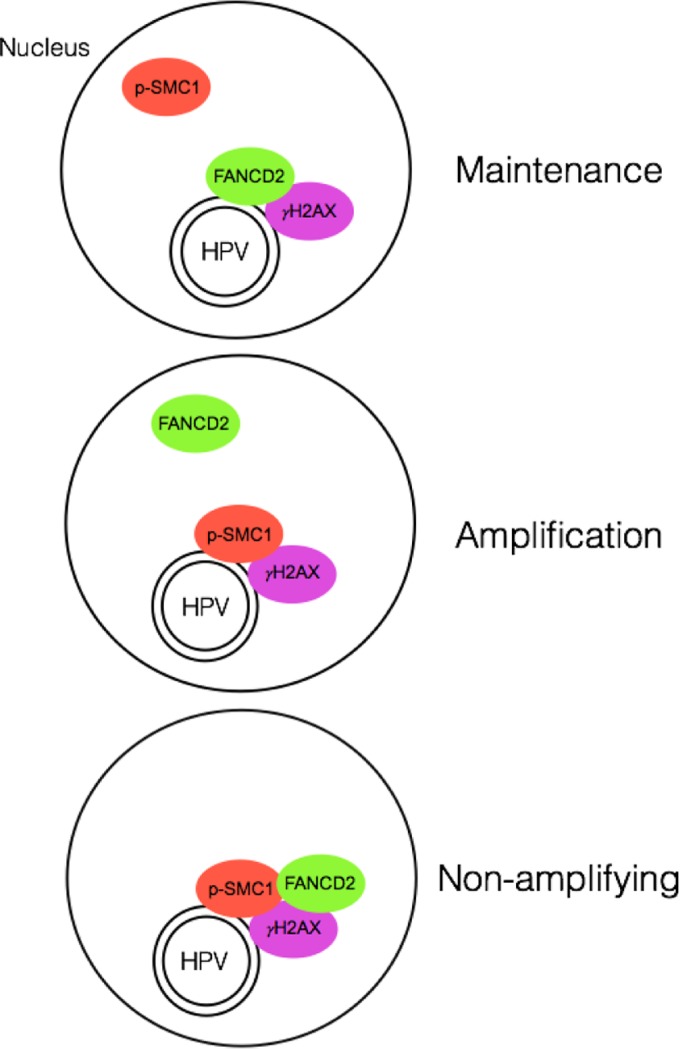
Model showing the different populations of FANCD2/γH2AX/HPV DNA and p-SMC1/ γH2AX/HPV DNA foci found in HPV-positive cells upon differentiation. p-SMC1 and FANCD2 are rarely found together in the same nuclei. The cells with p-SMC1/γH2AX bound to HPV genomes likely represent the amplifying population, while those with FANCD2/γH2AX are not amplifying.

Previous studies have shown that high-risk HPVs activate the ATM and ATR DNA damage response pathways and that members of these pathways, such as γH2AX, BRCA1, and p-SMC1, colocalize to distinct nuclear foci that also contain viral genomes (37, 38). In the present study, we found that these factors were not all found in the same foci as FANCD2 but were present in different sets of foci. In particular, we observed that FANCD2 preferentially colocalizes with γH2AX and BRCA1, but at significantly reduced levels with p-SMC1. Using 4-color immunofluorescence, we discovered that at least three distinct populations of cells exist in HPV-positive cells. FANCD2 is found with p-SMC1 in nuclear foci of approximately 10 to 20% of cells, while it is colocalized with BRCA1 and γH2AX in foci of over 80% of cells. p-SMC1 also localizes with γH2AX in foci, but in a different population of cells than those containing FANCD2. These distinct populations of foci observed in HPV-positive cells undergoing differentiation suggest that these proteins have different roles in viral replication and amplification. A model is shown in Fig. 9.

Chromatin immunoprecipitation assays demonstrate that FANCD2 binds to viral genomes in undifferentiated cells, and this binding decreases by 3- to 6-fold upon differentiation. Interestingly, the level of FANCD2 binding to viral genomes was significantly higher than that to sequences in the host chromosome, including at least one fragile site (FRA3B). The level of FANCD2 binding to viral genomes was, however, similar to binding at another fragile site (FRA16D), suggesting that FANCD2 is being recruited to HPV genomes either to repair damage to viral DNA or for another mechanism that is not yet fully understood. Similar localization of FANCD2 to viral genomes was seen in immunofluorescent *in situ* hybridization (I-FISH) assays for FANCD2 at HPV31 replication foci. In undifferentiated cells, FANCD2 and HPV DNA signals substantially overlapped in small, defined foci. In contrast, upon differentiation, multiple large HPV31 foci appeared, but FANCD2 localized with only a subset.

Since our studies demonstrated FANCD2 binding to viral genomes, it was important to determine whether FANCD2 played any role in viral replication. Knockdown of FANCD2 with two individual shRNAs resulted in a rapid loss of viral episomes in undifferentiated cells and reduced genome amplification in differentiated cells. Interestingly, the fold amplification values were similar between knockdown and control cells, indicating that the reduced levels of genome amplification observed were the result of lower starting levels of episomes in undifferentiated cells. In addition, Northern blot analysis revealed that FANCD2 does not have an effect on early viral gene expression and therefore does not impair episomal maintenance by reducing the expression of viral replication proteins. It is anticipated that following long-term passage of FANCD2 knockdown cells, this loss of episomal maintenance would lead to increased viral genome integration into host DNA, although these experiments have not yet been performed. These studies indicate that FANCD2 plays an important role in regulating the stable maintenance of HPV episomes in undifferentiated cells, but has a less significant effect on amplification.

Previously, we observed that knockdown of SMC1 had a significant effect on genome amplification, identifying it as a critical regulator in this process (37). As discussed, our studies suggest that distinct populations of repair proteins are present in differentiating HPV-positive cells—one set of cells containing p-SMC1-positive foci and another with FANCD2. As only a subset of HPV-positive cells amplify viral genomes, we believe that the cells containing p-SMC1 foci represent actively amplifying cells and not the cells expressing FANCD2. This is further supported by immunofluorescent *in situ* hybridization data showing that p-SMC1 colocalizes with HPV DNA in differentiated cells.

HPV activation of the ATM pathway is required for viral genome amplification in differentiated cells, but has no effect on episomal maintenance in undifferentiated cells (13). Stable as well as transient replication of HPV genomes induces an ATR response that leads to the accumulation of ATR pathway members at viral replication centers (14, 15). Importantly, inhibition of CHK1, the downstream target of ATR, leads to a reduction in the stable maintenance of genomes in undifferentiated cells (15). Our studies show that knockdown of FANCD2 also leads to a reduction in episomal copy number in undifferentiated cells, suggesting that the ATR pathway regulates HPV replication in undifferentiated cells, at least in part, through the FA pathway. This is consistent with reports that the phosphorylation of FA proteins by ATR and CHK1, including FANCD2, is required for FA pathway activation in response to DNA damage (47).

Previous studies reported a hyperplastic phenotype in FA cells, which is specific to an HPV-positive environment (31, 32). We observed a similar growth phenotype in our knockdown cells in culture as well as when grown in organotypic rafts. This suggests that the hyperplastic phenotype seen with FA deficiency in HPV-infected epithelia is not dependent on the presence of viral episomes. In addition, a previous study demonstrated that knockdown of FANCD2 led to increased levels of viral genomes upon differentiation (31), which contrasts with our observations. We are investigating the source of these differences, which may be related to the enhanced appearance of multimeric genomes observed upon differentiation or differences in methods used.

Fanconi anemia patients have an inherent susceptibility to HPV-associated malignancies, suggesting that the loss of FA pathway activity promotes oncogenesis (48); however, the role that the FA pathway plays during viral infection is unclear. Previous studies found that HPV16 E7 can induce head and neck SCCs in FANCD2 knockout mice and that the loss of either FANCD2 or the FA core component FANCA stimulates the posttranscriptional accumulation of the E7 viral oncogene in keratinocytes (31, 49). We suggest a model in which FANCD2 is recruited to HPV DNA, where it colocalizes with and recruits other DNA repair proteins to viral replication centers. This occurs either through the presence of interstrand cross-links in viral DNA or, possibly, through the action of a viral protein. This recruitment allows for the efficient and faithful replication of viral episomes in basal epithelial cells. In the absence of FA pathway activation, as seen in FA patients, FANCD2 is not recruited to host or viral genomes, leading to increased genomic instability, the loss of episomal maintenance, and, likely, increased integration into the host’s genome. Integration results in enhanced expression of viral oncogenes in cells, which can result in an increased susceptibility to cancer. Overall, our studies identify the FA pathway as a key regulator of viral replication in basal replicating cells and further illustrate how HPV promotes carcinogenesis in FA patients.

## MATERIALS AND METHODS

### Cell lines.

Human foreskin keratinocytes (HFKs) were isolated from deidentified neonatal foreskin and grown as previously described (50). HFKs containing HPV31 (HFK31) were generated by cotransfecting recircularized HPV31 genomes (pBR-322min) and an antibiotic resistance plasmid (pSV2 Neo) using FuGene6 (Promega) into HFKs followed by selection with G418 (Sigma). HFK16 cells were generated as described previously (51). CIN612 cells were obtained from a patient biopsy specimen of a low-grade cervical neoplasia (52). All cell lines were cultured in E-medium supplemented with mouse epidermal growth factor (EGF) (53) and maintained on mitomycin C-treated J2 fibroblast feeder cells (54).

### Calcium-induced differentiation.

Cells were grown to 80% confluence in E-medium with EGF and switched to M154 medium supplemented with human keratinocyte growth supplement (Invitrogen), penicillin, streptomycin, and 0.03 mM filter-sterilized calcium chloride. After 24 h, medium was replaced with M154 containing 1.5 mM calcium chloride. Cells were allowed to differentiate for 48 or 72 h in high-calcium medium.

### Methylcellulose-induced differentiation.

To induce differentiation, between 3 × 10^6^ and 6 × 10^6^ cells were suspended in E-medium containing 1.5% methylcellulose and allowed to grow for 24 or 48 h. Cells were then harvested by centrifugation following two washes in cold phosphate-buffered serine (PBS) (55).

### Western blot analysis.

Whole-cell lysates were extracted using radioimmunoprecipitation assay (RIPA) lysis buffer (50 mM Tris [pH 7.4], 150 mM sodium chloride [NaCl], 0.25% deoxycholic acid, 1% NP-40, 1 mM EDTA) supplemented with protease inhibitor cocktail (Roche). Protein from the insoluble fraction was extracted from the cell pellet using a solubilization solution (8 M urea, 10% 2-mercaptoethanol, 2 mM phenylmethylsulfonyl fluoride [PMSF]) and incubated at 37°C for 30 min. Protein was quantitated using a Bradford assay (Bio-Rad) and run on a Tris-glycine sodium dodecyl sulfate (SDS)-polyacrylamide gel. Protein was transferred to an Immobilon-P polyvinylidene fluoride membrane (Millipore) and probed with primary and secondary antibodies. ECL (enhanced chemiluminescence) or ECL Prime (GE) was used to visualize protein. The antibodies used were FANCD2 (Abcam no. ab108928; Thermo Scientific catalog no. MA1-16570), cytokeratin 10 (Santa Cruz catalog no. sc52318), γH2AX-Ser139 (Cell Signaling catalog no. 5438), FANCI (Santa Cruz catalog no. sc-98532), BRCA1 (Cell Signaling catalog no. 9010), RAD51 (Cell Signaling catalog no. 8875), BRCA2 (Cell Signaling catalog no. 9012), and GAPDH (glyceraldehyde-3-phosphate dehydrogenase; Santa Cruz catalog no. sc-47724).

### Immunofluorescence.

Cells were grown on no. 1 glass coverslips and fixed with 4% methanol-free paraformaldehyde (PFA) in PBS. Cells were then permeabilized in PBS plus 0.1% Triton X-100 (PBT) and blocked with normal goat serum plus 0.15 Triton X-100 (NGS-T) followed by incubation with primary antibody in NGS-T overnight at 4°C in a humidity chamber. Coverslips were washed in PBS and incubated with Alexa Fluor secondary antibody (Invitrogen) for 30 min at 37°C. After PBS washes (3×), cells were counterstained with DAPI (4′,6-diamidino-2-phenylindole) and mounted with Gelvatol. The antibodies used were FANCD2 (Novus Biologicals, Inc., catalog no. NB100-182), BRCA1 (Oncogene catalog no. OP92), γH2AX-Ser139 (Millipore catalog no. 05-636), and p-SMC1-Ser957 (Cell Signaling catalog no. 4805). For 4-color immunofluorescence, following secondary washes, cells were incubated with Alexa Fluor 647-γH2AX-pS139 (BD Pharmingen no. 560447) in a light-protected humidity chamber for 1 h at room temperature. Coverslips were washed in PBS, counterstained with DAPI, and mounted in Gelvatol. Cells were imaged using a Zeiss Axioscope and analyzed using ImageJ.

### ImageJ automated particle analysis.

Images were taken using a Zeiss Axioscope and imported into ImageJ to quantitate focus size. Images were converted to 8 bits, and the threshold was adjusted to 185 to 255. The image was then processed as binary, and a watershed was applied. Large FANCD2 foci were characterized as particles between 1 and 75 in^2^, displayed in pixel units (*n* ≥ 221 cells).

### ChIP.

Cells were grown to 80% confluence and cross-linked with 1% formaldehyde for 8 min. The reaction was quenched with 2.5 M glycine (125 mM final) followed by at least three washes in ice cold PBS. Cells were collected and resuspended in lysis buffer 1 (50 mM HEPES, 140 mM NaCl, 1 mM EDTA, 10% glycerol, 0.5% NP-40, 0.25% Triton X-100) supplemented with protease inhibitor. Cells were then pelleted and resuspended in lysis buffer 2 (10 mM Tris HCl, 200 mM NaCl, 1 mM EDTA, 0.5 mM EGTA). Cells were again pelleted and resuspended in lysis buffer 3 (10 mM Tris-HCl, 10 mM NaCl, 1 mM EDTA, 0.5 mM EGTA, 0.1% sodium deoxycholate, 0.5% Sarkosyl), and chromatin was sheared using a Bioruptor ultrasonic bath (Diagenode). Lysate was incubated with 2 μg of antibody to FANCD2 (Novus Biologicals, Inc., catalog no. NB100-182), γH2AX-Ser139 (Millipore catalog no. 05-636), or an IgG control overnight at 4°C. The next day, protein G Dynabeads (Life Technologies, Inc.) were washed in lysis buffer 3, added to the lysate-antibody mixture, and allowed to rotate for 2 to 3 h at 4°C. Beads were collected and washed several times in wash buffer (50 mM HEPES, 50 mM lithium chloride, 1 mM EDTA, 1% NP-40, 0.7% sodium deoxycholate). Chelex 100 suspension (Bio-Rad) was added to the beads, and the mixture was boiled for 10 min at 95°C. After cooling, the tubes were incubated with proteinase K for 30 min at 55°C. Proteinase K was inactivated by again boiling the beads at 95°C, and DNA was collected following centrifugation. Real-time touchdown PCR was performed with the LightCycler 480 (Roche) against primers listed in Table S1 in the supplemental material.

10.1128/mBio.02340-16.3 TABLE S1  List of forward (F) and reverse (R) primers used for chromatin immunoprecipitation (ChIP) assays. All primer sequences are shown in the 5′→3′ direction. Download TABLE S1, DOCX file, 0.1 MB. Copyright © 2017 Spriggs and Laimins. 2017 Spriggs and Laimins This content is distributed under the terms of the Creative Commons Attribution 4.0 International license.

### I-FISH.

Cells were grown on no. 1 glass coverslips and fixed with 4% methanol-free PFA before being permeabilized in PBT. Cells were then blocked with NGS-T and incubated with primary antibody overnight at 4°C in a humidity chamber. Coverslips were washed in PBS (3×) and incubated with secondary antibody. Cells were then treated with ice-cold methanol-acetic acid followed by 2% PFA. Coverslips were treated with RNase-It (Stratagene), dehydrated with 70, 85, and 100% ethanol, and dried for several hours. HPV31 probe (Enzo) in hybridization buffer (Empire Genomics) with Cot1 DNA was added to coverslips, denatured at 75°C, and hybridized overnight at 37°C. Coverslips were washed in wash buffer (0.5× saline-sodium citrate [SSC], 0.1% SDS) followed by a wash in phosphate-buffered detergent. Tyramide signal amplification was performed using TSA kit no. 22 (Life Technologies, Inc.). Cells were counterstained with DAPI and mounted in Gelvatol.

### Lentiviral knockdown.

Mission pLKO.1 shRNA targeting either GFP or FANCD2 (Sigma) was transfected into 50% confluent 293T cells, along with pVSVG and pGag-Pol-Tat-Rev, using X-tremeGENE HP DNA transfection reagent (Roche). Medium was changed 24 h posttransfection, and cells were allowed to grow for an additional 24 h. Viral supernatants were collected and concentrated using an Amicon centrifugal filter (Millipore). For lentiviral transduction, viral particles were incubated with target cells and Polybrene (8-μg/ml final concentration). Medium was changed 24 h posttransduction, and cells were allowed to grow for an additional 24 h. Cells were then either harvested, differentiated, or selected for stably silenced cell lines using puromycin. Knockdown was confirmed by Western blot analysis.

### Southern blot analysis.

Cells were collected and resuspended in Southern lysis buffer (400 mM NaCl, 10 mM Tris-HCl, [pH 7.4], 10 mM EDTA) and treated with RNase (50 μl/ml final), proteinase K (50-μl/ml final concentration), and 0.2% SDS. Total DNA was isolated by phenol-chloroform extraction and run on a 0.8% agarose gel. DNA was transferred to a membrane using a vacuum and probed with ^32^P-labeled HPV31 DNA. The membrane was washed with SSC/SDS wash buffer of various stringencies (2× SSC–0.1% SDS, 0.5× SSC–0.1% SDS, 0.1× SSC–0.1%, 0.1× SSC–1.0%) and analyzed by autoradiography (11).

### Northern blot analysis.

Total RNA was isolated using STAT60 (Tel-Test, Inc.) and run on a 1% gel containing 6% formaldehyde. RNA was transferred to a membrane using a vacuum and probed with ^32^P-HPV31 DNA. Following hybridization, membrane was washed twice in high-stringency wash buffer (1 mM EDTA, 40 mM Na_2_HPO_4_, and 5% SDS then 1% SDS) and analyzed by autoradiography (11).

### Organotypic raft culture.

Collagen gels containing J2 fibroblast feeder cells were prepared from a mix of rat tail collagen type 1 (BD Biosciences), 10× reconstitution buffer (2.2 g NaHCO_3_, 4.8 g HEPES in 100 ml 0.05 M NaOH), and 10× Dulbecco’s modified Eagle’s medium (DMEM) without NaHCO_3_. Gels were allowed to solidify in a 6-well cell culture dish for at least 1 h. Between 1 × 10^6^ and 2 × 10^6^ control or knockdown cells were seeded onto the top of a collagen gel and grown to confluence in E-medium with EGF. At confluence, E-medium was removed, and the collagen gel was transferred onto a metal grid in a 100-mm dish. An air-liquid interface was created by adding E-medium without EGF to the bottom of the dish so that it touches the metal grid but not the collagen. Rafts were incubated at 37°C, and medium was changed every other day. Rafts were harvested after 14 days and fixed, and paraffin blocks were generated by the Skin Disease Research Center at Northwestern University. This was followed by hematoxylin and eosin (H&E) staining for analysis.
