# Supplementary figures and images for: FANCD2 Binds Human Papillomavirus Genomes and Associates with a Distinct Set of DNA Repair Proteins to Regulate Viral Replication
Source: mBio. 2017 Feb 14;8(1):e02340-16. doi: 10.1128/mBio.02340-16 (PMC5312087; doi:10.1128/mBio.02340-16)

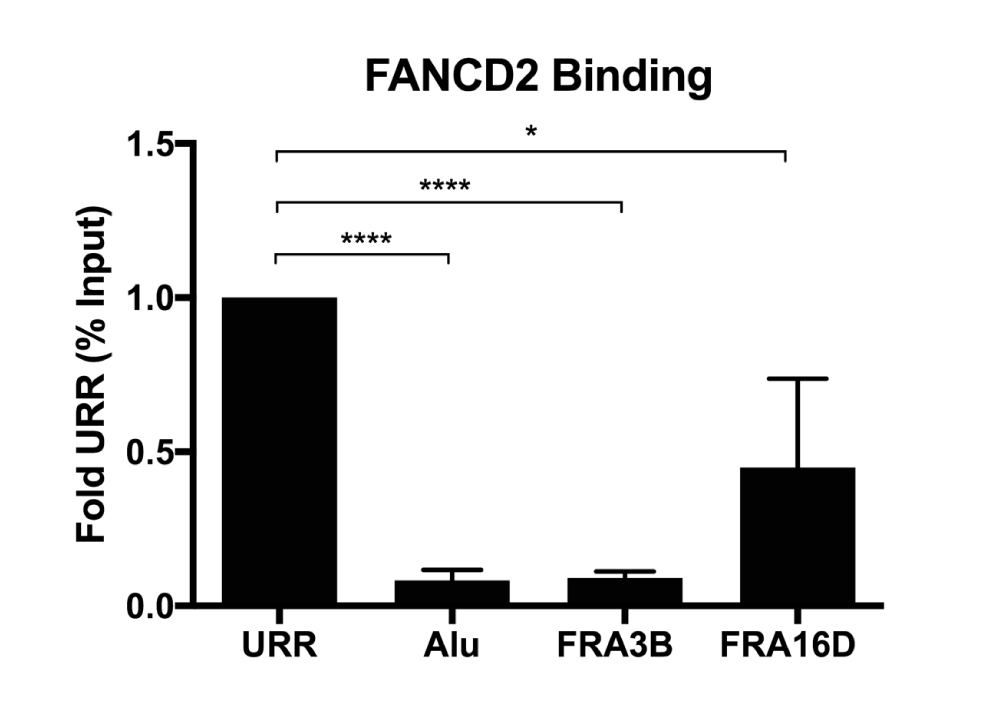

Supplement: FIG S1 [file mbo001173184sf1.tif]

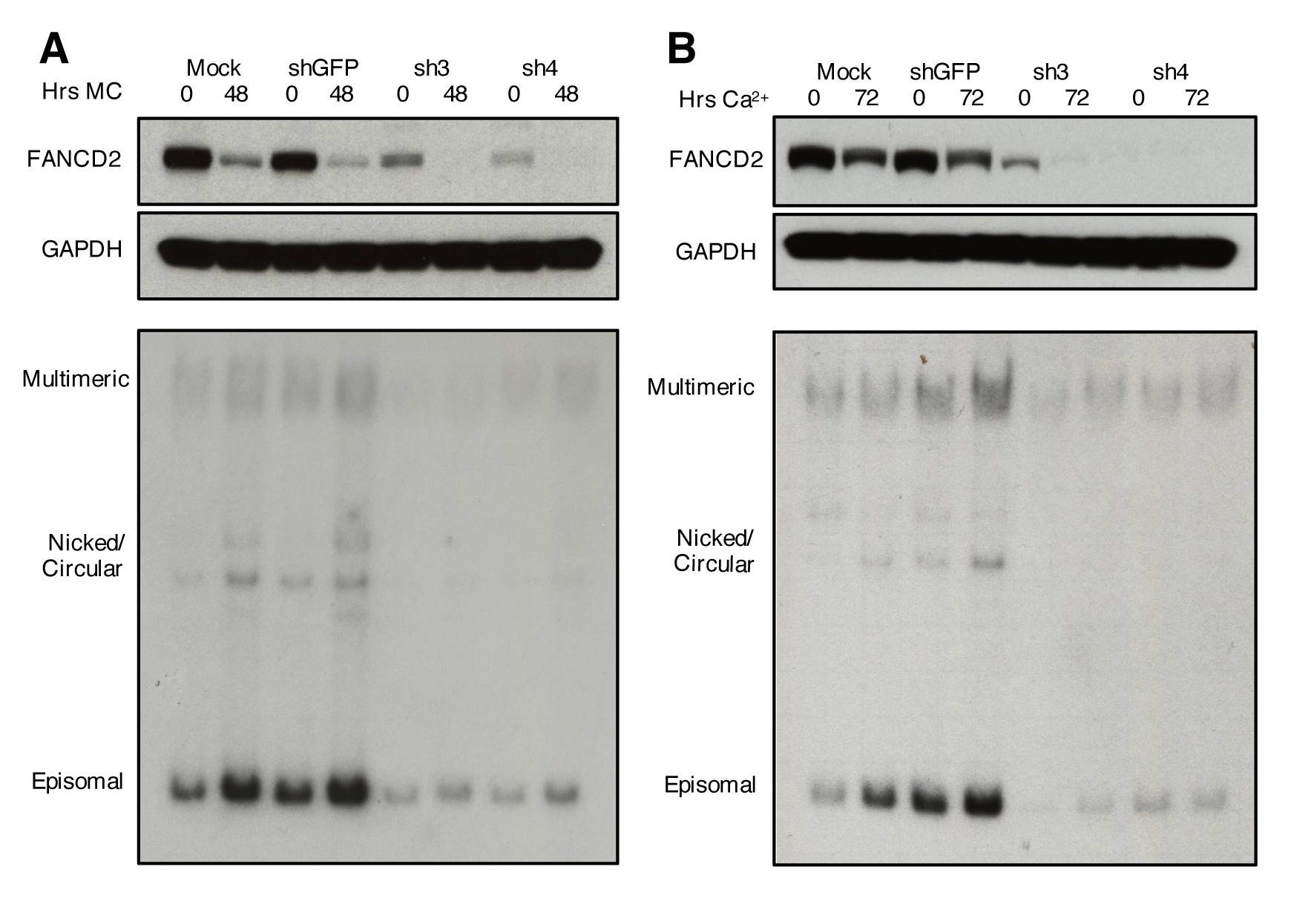

Supplement: FIG S2 [file mbo001173184sf2.tif]
