# Supplementary material for: FANCD2 Binds Human Papillomavirus Genomes and Associates with a Distinct Set of DNA Repair Proteins to Regulate Viral Replication
Source: mBio. 2017 Feb 14;8(1):e02340-16. doi: 10.1128/mBio.02340-16 (PMC5312087; doi:10.1128/mBio.02340-16)
Supplement: TABLE S1 [file mbo001173184st1.docx]

**Table S1** Primers used for Chromatin Immunoprecipitation (ChIP) assays.

| Primer Sequence |
| --- |
| URR F GATGCAGTAGTTCTGCGGTTT  URR R TATGTTGGCAAGGTGTGTTAGG  Late F ATGTCATAGACAGTCCAG  Late R AATTACTGCTTCTACATA  E7 F AATTACCCGACAGCTCAGATG  E7 R GGCACACGATTCCAAATGAG  E2 F TACTGTTGTGGAAGGGCAAG  E2 R TCCCAGCAAAGGATATTTCGTC  L2 F TTTGGTGGGTTGGGTATTGC  L2 R GTAGGAGGCTGCAATACAGATG  Alu F (1) ACGAGGTCAGGAGATCGAGA  Alu R CTCAGCCTCCCAAGTAGCTG  FRA13B F (2) TGTTGGAATGTTAACTCTATCCCA  FRA13B R ATATCTCATCAAGACCGCTGCA  FRA16D F (2) TCCTGTGGAAGGGATATTTA  FRA16D R CCCCTCATATTCTGCTTCTA |

1. **Shah GA, O'Shea CC.** 2015. Viral and Cellular Genomes Activate Distinct DNA Damage Responses. Cell **162:**987-1002.

2. **Lu X, Parvathaneni S, Hara T, Lal A, Sharma S.** 2013. Replication stress induces specific enrichment of RECQ1 at common fragile sites FRA3B and FRA16D. Mol Cancer **12:**29.

**Supplementary References**
